# Coexistence of Pseudoangiomatous Stromal Hyperplasia and Benign Phyllodes Tumor: A Rare Histopathological Challenge

**DOI:** 10.7759/cureus.91441

**Published:** 2025-09-01

**Authors:** Hari Krishnan B, Jesu Pencilin Yesuvadiyan, Karthikeyan Selvaraj, S. Balakrishnan, Sasikumar Pattabi

**Affiliations:** 1 General Surgery, Sree Balaji Medical College and Hospital, Chennai, IND; 2 Surgery, Sree Balaji Medical College and Hospital, Chennai, IND; 3 Surgery, Bharath Institute of Higher Education and Research, Chennai, IND

**Keywords:** benign breast tumor, fibroepithelial lesion, pash, phyllodes tumor, pseudoangiomatous stromal hyperplasia, stromal proliferation, wide local excision

## Abstract

Phyllodes tumors are uncommon fibroepithelial breast neoplasms with potential for local recurrence and, rarely, malignant transformation. Pseudoangiomatous stromal hyperplasia (PASH) is a benign proliferative lesion of the breast stroma, usually encountered as an incidental microscopic finding but occasionally presenting as a mass-forming lesion. The coexistence of PASH within a phyllodes tumor is rare and can pose diagnostic challenges on core biopsy. We report the case of a 48-year-old multiparous woman who presented with a gradually enlarging left breast lump over two years. Clinical examination and imaging favored a phyllodes tumor. Fine-needle aspiration cytology (FNAC) and core biopsy, however, showed PASH-like features. Wide local excision was performed, and histopathology confirmed a benign phyllodes tumor with extensive PASH. The postoperative course was uneventful, and the patient remained asymptomatic at six months of follow-up. The presence of PASH-like features on core biopsy created a diagnostic dilemma, as the low stromal cellularity and pattern mimicked benign proliferative lesions, leading to an underestimation of the underlying fibroepithelial neoplasm. This underscores the importance of clinicoradiologic-pathologic correlation and surgical excision for definitive diagnosis in such cases.

## Introduction

Phyllodes tumors are uncommon biphasic fibroepithelial neoplasms, accounting for 0.3% to 1% of all breast tumors and about 2.5% of all fibroepithelial lesions of the breast [1,2]. These tumors are characterized by a proliferation of both epithelial and stromal components, and they are classified into benign, borderline, and malignant categories based on histologic features such as stromal cellularity, atypia, mitotic activity, and margin appearance [3]. Pseudoangiomatous stromal hyperplasia (PASH), on the other hand, is a benign mesenchymal lesion composed of anastomosing slit-like spaces lined by spindle cells within a collagenous stroma. These channels mimic vascular spaces but lack endothelial cells, thus distinguishing PASH from low-grade angiosarcoma [4-6].

The etiology of PASH remains unclear, but hormonal influence, particularly progesterone, is considered significant in its pathogenesis, explaining its frequent presentation in premenopausal women and those on hormone therapy [7,8]. While PASH typically presents as an incidental microscopic finding, it may occasionally form palpable masses or be detected radiologically. The coexistence of PASH with phyllodes tumors is rarely reported in the literature and may result in diagnostic confusion [9,10]. Previous studies have documented only a limited number of such cases, most of which describe incidental identification of PASH within excised phyllodes tumors, underscoring its rarity [11-13]. Phyllodes tumors themselves most commonly present in women between 35 and 55 years, whereas PASH typically occurs in premenopausal women, further complicating the diagnostic process when these two entities coexist [7,8,11]. In this report, we present a case of a middle-aged woman with a slowly enlarging breast mass, which on imaging and core biopsy suggested a fibroepithelial lesion, with histological features such as stromal hypercellularity and slit-like spaces overlapping between phyllodes tumor and PASH, thereby complicating the preoperative distinction between the two. We aim to emphasize the need for surgical excision and histological assessment for an accurate diagnosis in such complex presentations.

## Case presentation

**Figure 1:**
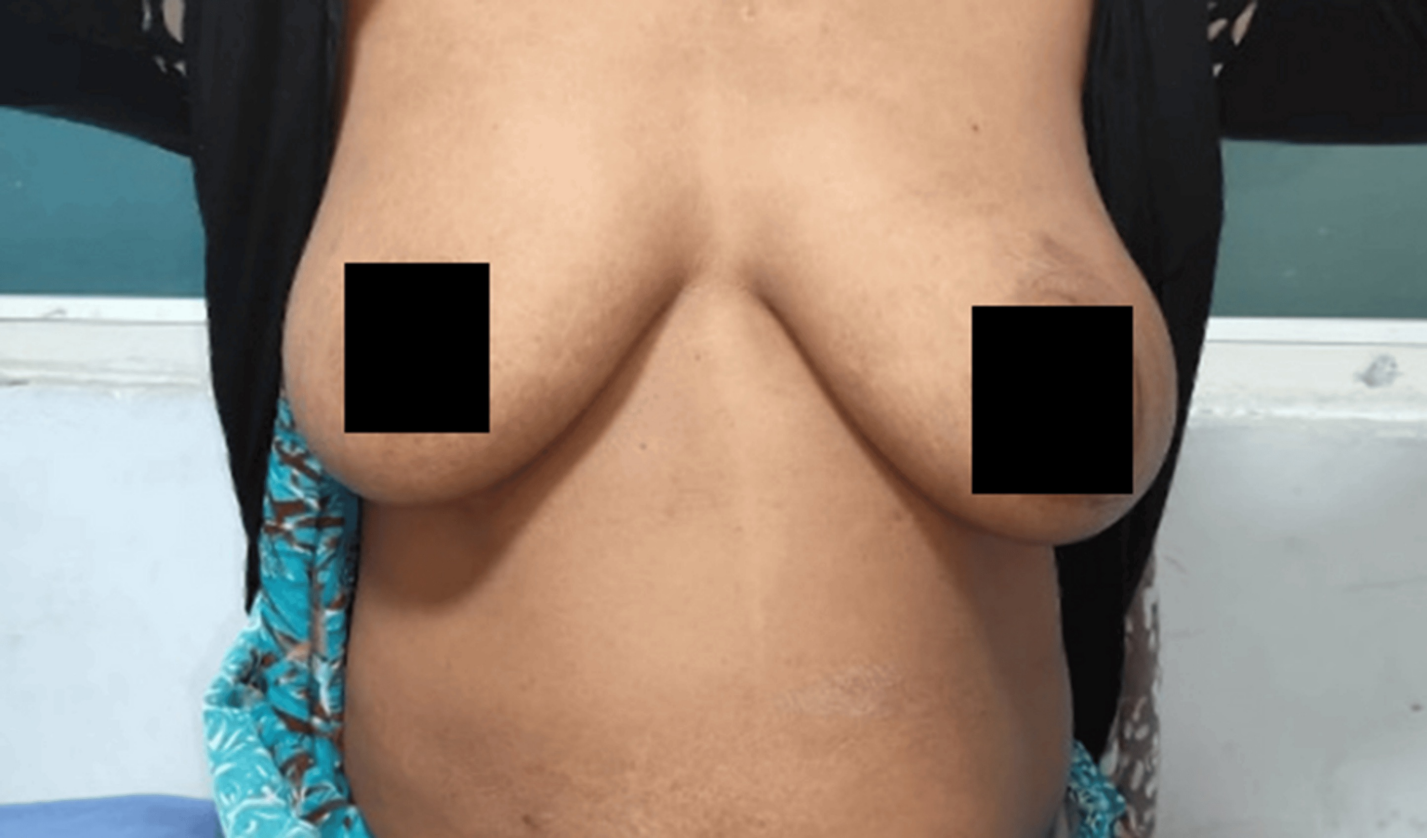
Clinical image of the left breast lump

A 48-year-old postmenopausal woman presented to the surgical outpatient department with complaints of a gradually enlarging, painless lump in the left breast for the past two years. She denied any history of nipple discharge, retraction, or systemic symptoms such as weight loss or fever. She had no significant family history of breast cancer. Obstetric history revealed two full-term vaginal deliveries, and she had breastfed both children for over a year. On clinical examination, a 10 × 6 cm globular mass was palpable, involving the upper outer, lower outer, and central quadrants of the left breast. The overlying skin was stretched with prominent superficial veins, but no ulceration or dimpling was noted. The mass was firm to hard, mobile, and not fixed to the underlying muscle. There was no axillary lymphadenopathy. The contralateral breast and axilla were normal, as shown in Figure 1.

Ultrasound revealed a well-circumscribed, oval, heteroechoic lesion measuring approximately 9.7 × 3.8 cm spanning from the 12 to 6 o'clock position. The lesion showed peripheral vascularity with minimal internal flow on Doppler, raising suspicion of a phyllodes tumor (BIRADS V). FNAC was inconclusive, showing a fibroepithelial lesion with spindle cells suggestive of PASH-like areas. The benign nature of the Tru-cut biopsy findings favored breast conservation and supported the choice of wide local excision.

**Figure 2:**
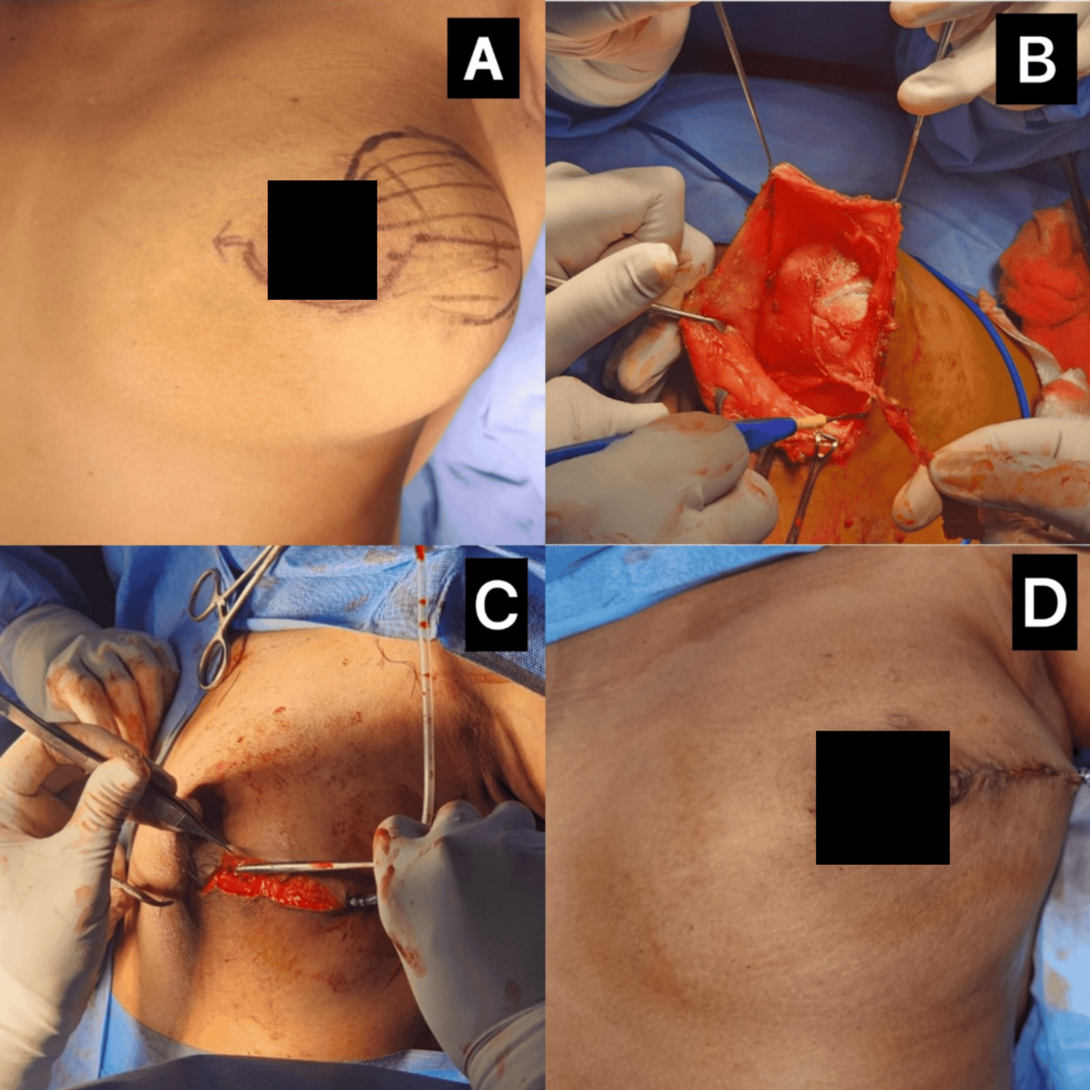
Intraoperative and postoperative images of wide local excision of phyllodes tumor (A) Preoperative marking for wide local excision using a circumareolar incision with lateral extension. (B) Intraoperative view showing the excised lump within the surgical cavity, with skin flaps retracted to expose the tumor. (C) Insertion of a suction drain following complete excision and hemostasis. (D) Immediate postoperative image showing a well-approximated incision with minimal skin puckering and preserved nipple-areola complex, indicating successful closure following wide local excision.

Although the tumor measured 10 cm, a wide local excision was feasible as adequate margins could be obtained. The patient’s preference for breast conservation also influenced the surgical plan, with mastectomy reserved as a secondary option if clear margins were not achievable. Given the imaging findings and progressive nature of the lesion, wide local excision was performed (Figures 2A-2D) using a circumareolar incision extended laterally. Grossly, the mass measured 10 × 6 cm, was firm in consistency, and was well-demarcated. The frozen section suggested a fibroepithelial lesion in favor of a proliferative breast disease. Given the lack of systemic symptoms and axillary lymphadenopathy, axillary sampling or dissection was not deemed necessary, consistent with the very low likelihood of nodal metastasis in phyllodes tumors. Intraoperatively, low-grade angiosarcoma was considered in the differential diagnosis; however, frozen section did not show endothelial lining of vascular spaces or significant cytologic atypia, thereby supporting a benign fibroepithelial lesion.

**Figure 3:**
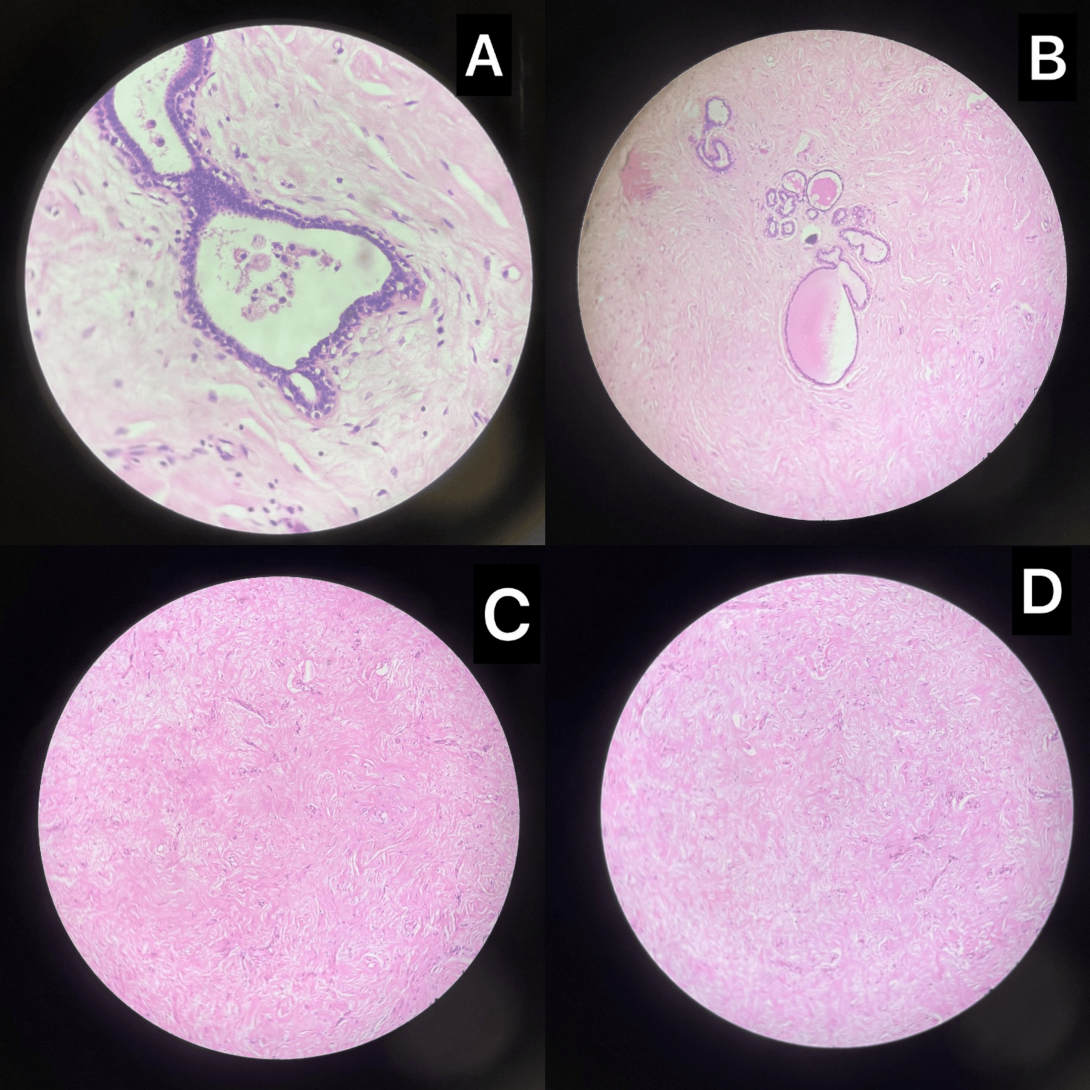
Histopathological features of benign phyllodes tumor with incidental pseudoangiomatous stromal hyperplasia (PASH) (A) Ducts containing foamy macrophages within fibrous stroma (hematoxylin and eosin (H&E) stain, ×100 magnification). (B) Dilated ducts lined by benign bilayered epithelium (H&E stain, ×100 magnification). (C) Areas of stromal hyalinization with reduced cellularity (H&E stain, ×100 magnification). (D) PASH showing slit-like pseudovascular spaces lined by myofibroblasts (H&E stain, ×100 magnification).

Final histopathology revealed a benign phyllodes tumor with extensive areas of PASH (Figure 3). The slit-like spaces lined by myofibroblastic cells were CD34-positive and CD31-negative on immunohistochemistry, confirming the diagnosis of PASH. No mitoses, atypia, or malignant transformation were noted.

**Figure 4:**
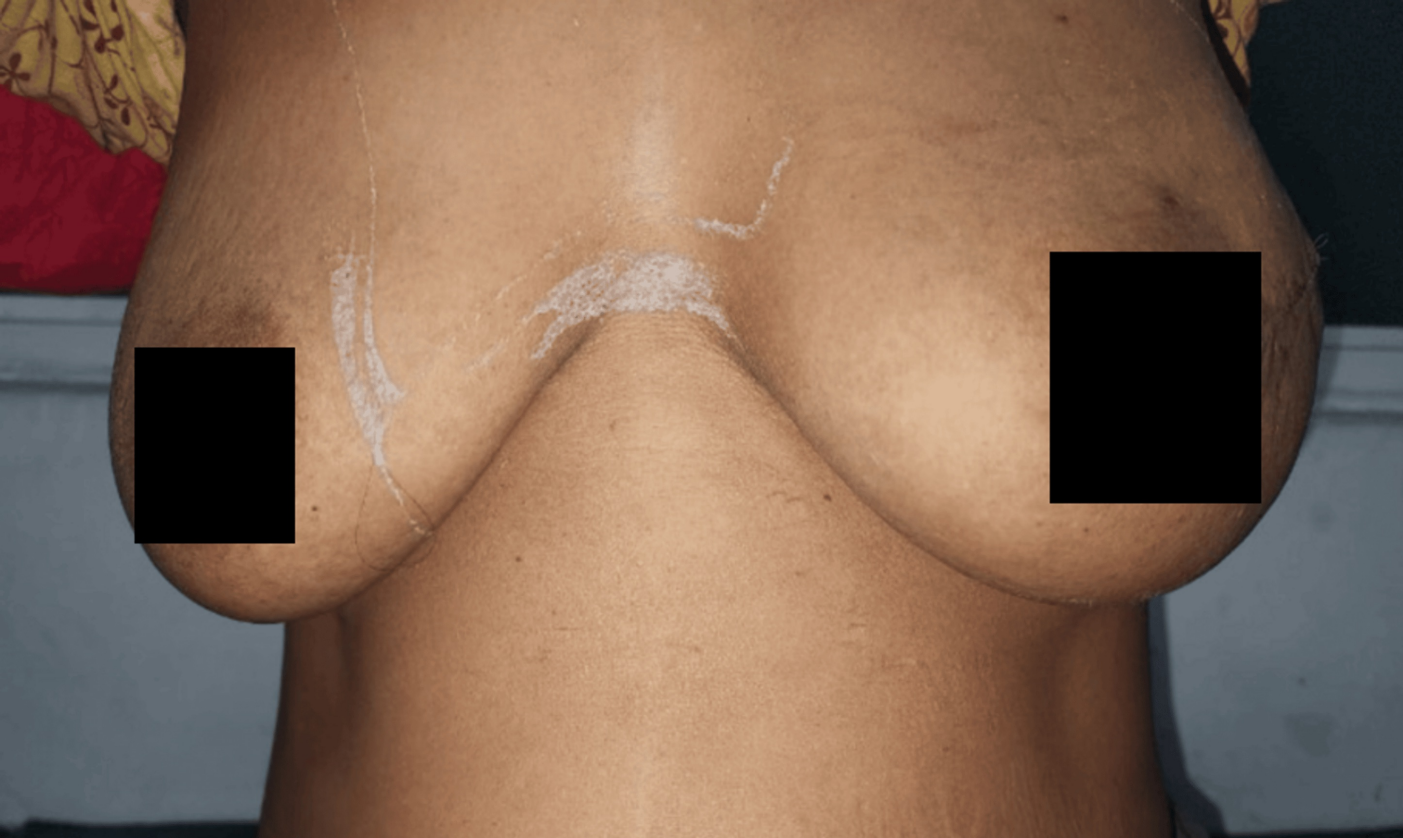
Postoperative day 14 follow-up Clinical photograph on postoperative day 14 showing a well-healed surgical scar on the left breast with restored breast symmetry. No signs of wound infection, seroma, or hematoma. The nipple-areola complex is intact, and the cosmetic outcome is satisfactory following wide local excision of a phyllodes tumor.

The patient recovered uneventfully and was discharged on the second postoperative day. As shown in Figure 4, the patient was reviewed on postoperative day 14 following wide local excision of a phyllodes tumor with incidental PASH at the six-month follow-up; she remained asymptomatic with no evidence of recurrence. Although the patient remained asymptomatic with no recurrence at six months, longer follow-up is warranted due to the risk of delayed recurrence in phyllodes tumors. She continues to remain under regular surveillance at our institution. Postoperative imaging was not performed, as excision margins were histologically free of tumor. The patient is being monitored clinically, with imaging reserved for new symptoms or abnormal findings on examination.

## Discussion

Phyllodes tumors are rare fibroepithelial neoplasms of the breast comprising <1% of all breast tumors and typically affect women between 35 and 55 years of age [1]. They are classified into benign, borderline, and malignant based on histologic features such as stromal cellularity, atypia, mitotic activity, and margin status [2]. Although benign phyllodes tumors are the most common subtype, all variants require surgical excision with adequate margins to minimize the risk of recurrence or progression [3,4].

Histopathologically, phyllodes tumors exhibit a biphasic pattern with stromal overgrowth, leaf-like architecture, and compressed epithelial-lined ducts [5]. Imaging features are nonspecific and often mimic fibroadenomas, typically appearing as well-defined, lobulated, hypoechoic lesions on ultrasound or solid masses on mammography or MRI [6,7]. Fine-needle aspiration cytology (FNAC) and core needle biopsies can be inconclusive in differentiating phyllodes tumors from fibroadenomas due to overlapping cytologic features [8,9]. Thus, histopathology remains the gold standard for diagnosis and subclassification [10].

Surgical excision remains the cornerstone of treatment. Wide local excision with at least a 1 cm clear margin is recommended for all phyllodes tumors to reduce the risk of recurrence [3,11]. Local recurrence rates vary between 10% and 40%, depending on the tumor grade and adequacy of excision margins [12,13]. Although rare, malignant phyllodes tumors have metastatic potential, most commonly to the lungs and bones [14,15]. Axillary lymph node metastasis is uncommon and does not generally warrant dissection unless clinically indicated [10]. 

In our case, the histopathological examination also revealed incidental PASH, a benign stromal proliferation that can mimic vascular lesions. PASH typically occurs in premenopausal women or those on hormone therapy, but may also be seen incidentally in excised breast tissue [16]. On microscopy, it is characterized by interanastomosing slit-like spaces within a dense collagenous stroma, lined by spindle-shaped myofibroblasts without red blood cells. In our case, immunohistochemistry was performed on the excised mass, which demonstrated CD34 and SMA positivity and was negative for endothelial markers CD31 and Factor VIII, thereby confirming PASH [16].

The coexistence of PASH within phyllodes tumors is extremely rare, with only a few isolated case reports and small series described in the literature [13-16]. Most of these cases reported incidental detection of PASH within excised phyllodes tumors, highlighting the importance of thorough histological assessment. This limitation is compounded when PASH coexists with phyllodes tumor, as both can demonstrate stromal overgrowth and spindle cell proliferation, making them difficult to distinguish on limited biopsy samples. While phyllodes tumors mandate surgical management, PASH lesions may be observed or locally excised if symptomatic or enlarging [16]. The surgical margins in such cases are determined by the phyllodes tumor component, as PASH alone does not mandate wide excision. Therefore, the coexistence of PASH does not justify more aggressive margins beyond what is required for phyllodes tumors. For surgeons, the key implication is to ensure adequate excision margins guided by the phyllodes component, while pathologists must differentiate PASH from mimics such as low-grade angiosarcoma using histology and immunohistochemistry. The comparative features of these two entities are summarized in Table 1.

**Table 1:** Comparison of phyllodes tumor and PASH in clinical and histopathological features

Feature	Phyllodes tumor	Pseudoangiomatous stromal hyperplasia (PASH)
Lesion type	Biphasic fibroepithelial neoplasm	Benign stromal proliferation
Age group affected	35-55 years	30-50 years (often premenopausal)
Clinical presentation	Painless, rapidly enlarging palpable breast mass	Painless, slow-growing mass or incidental finding
Growth rate	Moderate to rapid	Usually slow
Histologic architecture	Leaf-like stromal projections; hypercellular stroma, compressed ducts	Anastomosing slit-like spaces in dense collagenous stroma
Immunohistochemistry	CD34+, variable Ki-67 and p53 expression in malignant forms	CD34+, SMA+, CD31, and Factor VIII negative
Malignant potential	Possible (in borderline/malignant types)	None
Management approach	Wide local excision with ≥1 cm margins	Observation or local excision if symptomatic/growing
Recurrence risk	Present, especially with inadequate margins	Very low
Imaging characteristics	Well-defined solid mass, may mimic fibroadenoma on USG/MRI	Nonspecific; hypoechoic mass or stromal thickening
Histopathology diagnostic role	Essential for subtype classification	Essential to exclude angiosarcoma and confirm diagnosis

## Conclusions

The coexistence of PASH within a phyllodes tumor, though rare, can complicate diagnosis on core biopsy due to overlapping histological features. In this case, surgical excision was crucial in resolving the diagnostic uncertainty and confirming both entities. More broadly, when biopsy findings are inconclusive in large or rapidly enlarging fibroepithelial breast lesions, excision guided by clinical suspicion and tumor behavior remains essential for diagnostic certainty and appropriate management. While isolated PASH may be managed conservatively, the possibility of a phyllodes tumor necessitates timely resection. This case highlights the importance of considering mixed pathologies, emphasizing that surgical decision-making should balance clinical suspicion, imaging, and growth characteristics to ensure accurate diagnosis, guide follow-up, and optimize patient care.
